# Impact of Nutrition Interventions During Pregnancy on Maternal and Neonatal Outcomes: A Systematic Review and Meta-Analysis

**DOI:** 10.7759/cureus.106187

**Published:** 2026-03-31

**Authors:** Sangeetha X, Moonjelly V Smitha, Rashmi R Das, Alwin Issac, Geeta Bhardwaj

**Affiliations:** 1 Department of Obstetrics and Gynaecological Nursing, MS Ramaiah Institute of Nursing Education and Research, Bengaluru, IND; 2 College of Nursing, All India Institute of Medical Sciences, Bhubaneswar, IND; 3 Department of Pediatrics, All India Institute of Medical Sciences, Bhubaneswar, IND; 4 College of Nursing, All India Institute of Medical Sciences, Bhopal, IND

**Keywords:** evidence-based medicine, maternal nutrition, neonatal outcomes, nutrition supplementation, pregnancy, pregnancy outcomes

## Abstract

Maternal undernutrition continues to be a significant contributor to adverse pregnancy outcomes in low- and middle-income countries. This systematic review and meta-analysis examined the effects of balanced nutrition supplementation during pregnancy on maternal and neonatal outcomes by synthesizing evidence from randomized controlled trials. Across the included studies, supplementation was associated with modest improvements in maternal weight gain and neonatal anthropometric measures, particularly birth weight and length. However, consistent benefits were not observed for outcomes such as preterm birth, stillbirth, miscarriage, or maternal mortality. A small reduction in neonatal head circumference was noted, but its clinical relevance remains uncertain. Overall, balanced nutrition supplementation appears to offer measurable benefits for maternal and fetal growth in undernourished populations, although the strength of the evidence varies, and further well-designed, context-specific trials are needed to clarify optimal implementation strategies.

## Introduction and background

Pregnancy is associated with significant physiological and metabolic changes that are essential for fetal development and the growth of maternal tissues. These changes substantially increase nutritional demands, making adequate dietary intake essential for placental development, fetal growth, and the maintenance of maternal health. Inadequate intake of energy and essential nutrients during pregnancy may disrupt these adaptive processes and negatively influence pregnancy outcomes [1,2].

Undernutrition is a substantial global public health problem, particularly affecting maternal and child health. In women of reproductive age, undernutrition, often reflected by a low BMI (<18.5), has been associated with reduced physical capacity, impaired immune function, increased susceptibility to infections, and elevated risks of adverse maternal outcomes [2,3]. Poor maternal nutritional status has been associated with a range of complications, including hypertensive disorders of pregnancy, metabolic disturbances, impaired lactation, and unfavorable birth outcomes. Maternal undernutrition can impair fetal growth, resulting in adverse outcomes such as low birth weight and preterm birth, as well as long-term consequences including impaired growth, increased susceptibility to chronic disease, and reduced survival [4-7].

The burden of maternal undernutrition is significantly higher in low- and middle-income countries, particularly across South Asian regions. A considerable proportion of women enter pregnancy with inadequate nutritional reserves, with anemia and underweight widely reported among pregnant women in India. Across low- and middle-income countries, 20-39% of women have a low BMI (<18.5 kg/m²), with South Asia reporting the highest prevalence (~24%), followed by Sub-Saharan Africa and Southeast Asia, which also have similarly high burdens. India is highlighted due to its substantial contribution to the regional and global burden, where anemia affects 58.7% of pregnant women aged 15-49 years, while 33.3% of women of reproductive age are malnourished and 42.2% begin pregnancy underweight [8-10]. These conditions substantially contribute to low birth weight and associated neonatal complications, highlighting the need for effective nutritional interventions during pregnancy.

Nutrition-focused interventions have been implemented to address maternal undernutrition and its consequences. These strategies include micronutrient supplementation, dietary diversification, food fortification, balanced protein-energy supplementation, and nutrition education. Among these, balanced protein-energy supplementation has been increasingly emphasized as a strategy to enhance maternal gestational weight gain and promote optimal fetal growth [2,10,11-13].

This systematic review and meta-analysis aimed to synthesize evidence from randomized controlled trials evaluating the effects of nutritional supplementation during pregnancy on maternal nutritional status and neonatal outcomes. Specifically, the review examined the impact of such interventions on maternal weight gain, neonatal anthropometric measures, and selected adverse pregnancy outcomes.

## Review

Methodology

The review protocol was prospectively registered with the International Prospective Register of Systematic Reviews (PROSPERO) under registration number CRD42021286997. The conduct and reporting of the review adhered to the Preferred Reporting Items for Systematic Reviews and Meta-Analyses (PRISMA) guidelines [14].

Search Strategy and Data Sources

A systematic and comprehensive search of the literature was performed to identify eligible studies published between January 1998 and December 2024. The following electronic databases were searched: MEDLINE (via PubMed), Embase, CINAHL, Web of Science, IndMED, and Google Scholar. The search strategy combined Medical Subject Headings (MeSH) terms and free-text keywords related to pregnancy, maternal nutrition, protein-energy supplementation, food supplementation, and pregnancy outcomes.

Boolean operators (“AND” and “OR”) were used to refine the search. Reference lists of eligible articles and relevant reviews were also manually screened to identify additional studies. The detailed search strategy is provided in Appendix 1. No restrictions were placed on geographic location. Only articles published in English were considered.

Eligibility Criteria

Types of studies: Only randomized controlled trials (RCTs) were included. Cluster-randomized and open-label RCTs were eligible if random allocation was clearly described.

Participants: The review included studies involving pregnant women, irrespective of parity or gestational age at enrolment. Studies focusing exclusively on women with HIV infection or those receiving supplementation only during the postpartum period were excluded.

Interventions: Eligible interventions consisted of nutrition supplementation during pregnancy, defined as food-based or dietary interventions intended to improve maternal nutritional status. These included balanced protein-energy supplements, supplementary feeding programs, food distribution initiatives, and dietary interventions for undernourished pregnant women. Eligible interventions were required to provide a minimum of 200 kilocalories per day, with protein contributing less than 25% of total energy intake, consistent with the definition of balanced protein-energy supplementation. Interventions included food distribution or supplementary feeding programs targeting pregnant women, particularly those who were underweight, and were designed to improve maternal weight gain, with or without accompanying dietary counselling.

Comparators: Control groups consisted of pregnant women receiving standard antenatal care, placebo, or routine nutritional advice without additional supplementation.

Outcome measures: The primary maternal outcomes assessed in this review were gestational weight gain and changes in BMI during pregnancy. Neonatal primary outcomes included birth weight, birth length, and head circumference. Secondary maternal outcomes comprised anemia, pregnancy-related complications, mode of delivery, and maternal mortality. Secondary neonatal outcomes included low birth weight, small-for-gestational-age birth, preterm birth, stillbirth, miscarriage, gestational age at delivery, neonatal mortality, and congenital anomalies. When multiple reports from the same trial were identified, the most comprehensive publication reporting relevant outcomes was included in the analysis.

Study Selection

**Figure 1:**
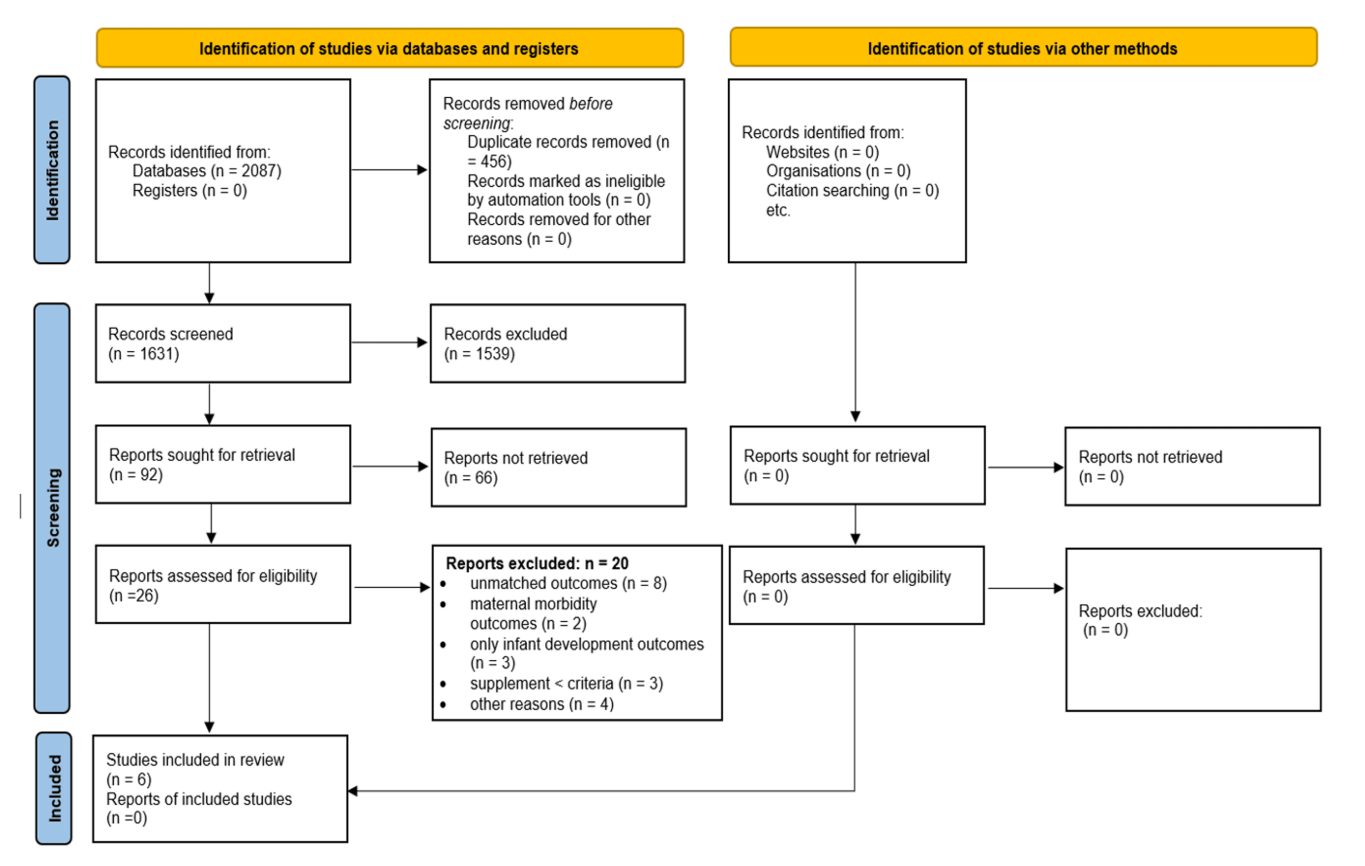
PRISMA 2020 flow diagram. PRISMA: Preferred Reporting Items for Systematic Reviews and Meta-Analyses.

All identified records were imported into a reference management system, and duplicates were removed. Initial screening of titles and abstracts was carried out independently by two reviewers to determine study eligibility. Full-text articles were retrieved for potentially relevant studies and assessed independently against the inclusion criteria. Any disagreements between reviewers were resolved through discussion, with the involvement of a third reviewer when required. The study selection process is illustrated using a PRISMA flow diagram (Figure 1).

Data Extraction

Data were extracted independently by two reviewers using a pre-designed and piloted data extraction form. Extracted information included study characteristics (author, year, country, design, setting), participant characteristics, details of the intervention and comparator, duration of follow-up, outcome measures, and funding sources (Table 1). Any discrepancies in the extracted data were resolved through consensus.

**Table 1:** Summary of data from all included studies. MUAC: Mid-Upper Arm Circumference; RCT: Randomized Controlled Trial.

Author, year [Reference]	Year of conduct of study, country	Study design, setting	Sample size (N), age of participants	Intervention (dose schedule)	Standard/common treatment used	Additional comments
Ceesay SM et al., (1997) [15]	1989-1994; Gambia	Multilevel RCT (2-arm); community setting	Randomized: 2047 (intervention = 1037, control = 1010); age: 15-45 years	Lipid-based supplementation; two biscuits daily	Iron-folic acid	The trial involved 2047 chronically undernourished women during a 5-year study period. The supplement provided during the hungry (wet) season (June to October) contained 22 g of protein and 1015 kcal. Compliance with the supplement was 100%. Funded study.
Qamar FN et al., (2017) [16]	2014-2016; Pakistan	Open-label RCT (2-arm); community setting	Randomized: 300 (*233; intervention = 150, control = 150); age: mean was 23 years	Lipid-based supplementation; one biscuit packet daily	Iron-folic acid	The BMI of included women was <19.9. The supplement provided 8 g of protein and 450 kcal. Compliance with the supplement was 75.3%. Funded study.
Hambidge KM et al., (2019) [17]	2013-2017; multi-country (India, Pakistan, Congo, Guatemala)	Women First Trial: cluster RCT (3-arm); community setting	Randomized: 7387 (*2124; intervention = 1029, control = 1095); age: 16-35 years	Lipid-based supplementation; one sachet daily	Iron-folic acid	The nutritional supplement delivered 2.6 g of protein and 118 kcal. Adherence to the supplementation regimen was greater than 87%. The study was funded.
Stevens B et al., (2018) [18]	2013-2015; Bangladesh	Cluster RCT (2-arm); community setting	Randomized: 87 (intervention = 58, control = 29); age: 14-31 years	Lipid-based supplementation; one sachet daily	Iron-folic acid	The supplement provided 19.5 g of protein and 522 kcal. Compliance with the supplement was 100%. Funded study.
Nga HT et al., (2020) [19]	2011-2015; Vietnam	VINAVAC study: RCT (3-arm); community setting	Randomized: 460 (*307; intervention = 150, control = 157); age: 18-30 years	Dark-green leafy vegetables and animal-source foods were used to prepare capsules that were given 5 days a week	Standard perinatal care	The supplement delivered ≥50% of the recommended dietary allowance for iron, zinc, folate, vitamin A, and vitamin B12. The attrition rate was 31%. A modified intention-to-treat analysis was performed. Funded study.
Hendrixson DT et al., (2020) [20]	2017-2020; Sierra Leone	Open-label RCT (2-arm); community setting	Randomized: 1489 (intervention = 752, control = 737); age: mean was 21 years	Lipid-based supplementation; daily ready-to-use supplementary food ration	Iron-folic acid	The included women were undernourished, with MUAC ≤23. The supplement provided 18 g of protein and 520 kcal. The intervention group received azithromycin in addition. Another supplement that provided 589 kcal and 17.5 g of protein was shared among the control group and their family. Both groups received prophylactic antimalarial drugs. Compliance with the supplement was ≥94%.

Risk of Bias Assessment

**Figure 2:**
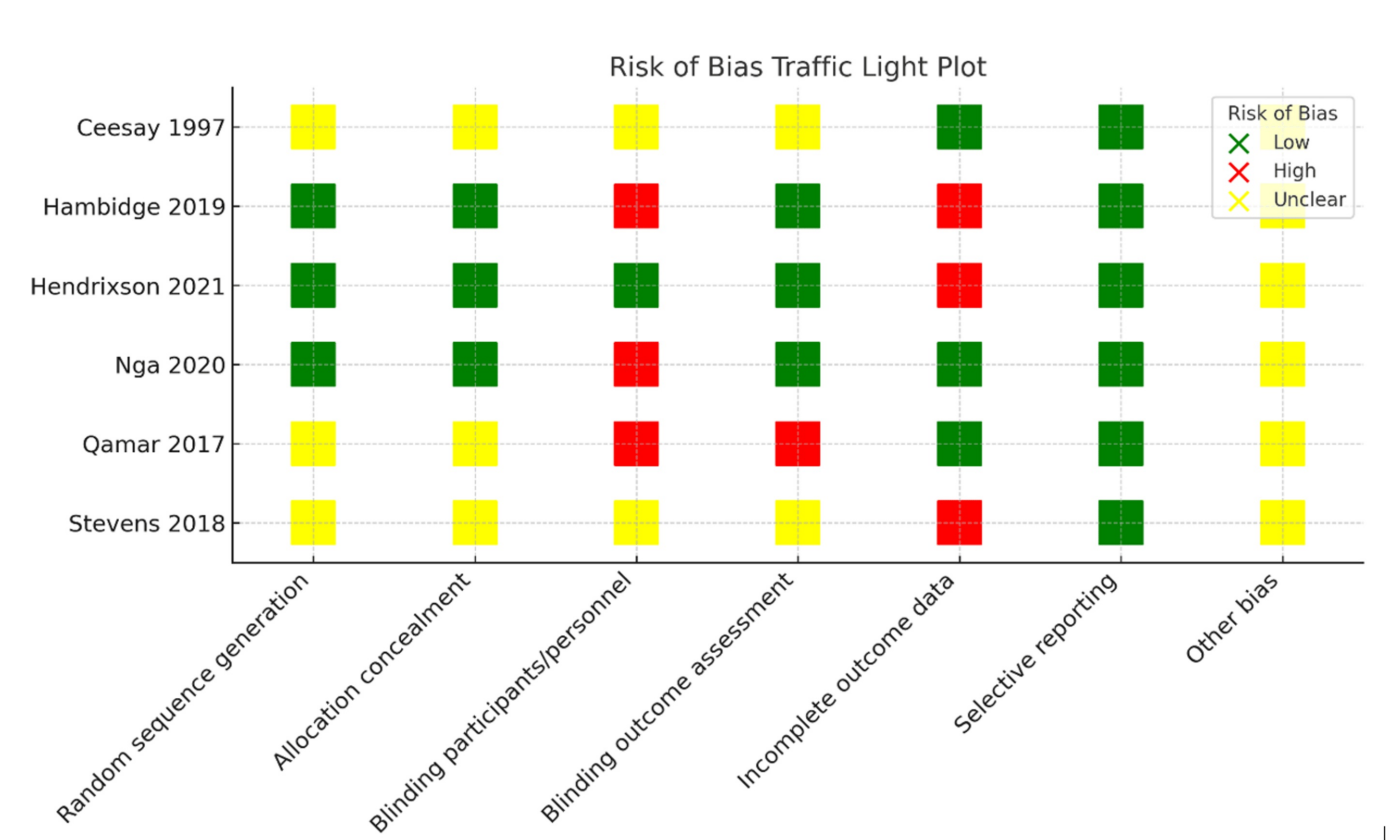
Risk of bias assessment of the included studies. Reference: Ceesay SM et al., (1997) [15]; Hambidge KM et al., (2019) [17]; Nga HT et al., (2020) [19]; Qamar FN et al., (2017) [16]; Stevens B et al., (2018) [18]; and Hendrixson DT et al., (2020) [20]. All figures included in this systematic review and meta-analysis were prepared by the authors and are original.

The methodological quality of the included studies was assessed using the Cochrane Risk of Bias Tool. The following domains were evaluated: random sequence generation, allocation concealment, blinding of participants and personnel, blinding of outcome assessment, selective outcome reporting, and completeness of outcome data. Two reviewers independently assessed the risk of bias, and disagreements were resolved through discussion. The results of the risk of bias assessment are summarized in Table 2 and Figure 2.

**Table 2:** Risk of bias among the included studies. ITT: Intention-to-treat analysis; SNOSE: Serially numbered, opaque, sealed envelopes.

Study [Reference]	Random sequence generation	Allocation concealment	Blinding of personnel and participants	Blinding of outcome assessors	Selective reporting	Missing data
Ceesay SM et al., (1997) [15]	Unclear	Unclear	Unclear	Unclear	Low	Low
Reason	Not described	Not described	Not described	Not described	No	ITT analysis
Qamar FN et al., (2017) [16]	Unclear	Unclear	High	High	Low	Low
Reason	Not described	Not described	No (open-label)	No (open-label)	No	No
Stevens B et al., 2018 [18]	Unclear	Unclear	Unclear	Unclear	Low	High
Reason	Not described	Not described	Not described	Not described	No	>35% attrition
Hambidge KM et al., 2019 [17]	Low	Low	High	Low	Low	High
Reason	Computer-generated	SNOSE	No (open-label)	Yes	No	>15% attrition
Nga HT et al., 2020 [19]	Low	Low	High	Low	Low	Low
Reason	Computer-generated	SNOSE	No (open-label)	Yes	No	Modified ITT analysis
Hendrixson DT et al., 2020 [20]	Low	Low	Low	Low	Low	High
Reason	Computer-generated	SNOSE	Yes	Yes	No	No ITT done

Assessment of Reporting Bias

Potential publication and selective reporting bias were assessed by examining trial registration records, the availability of study protocols, and consistency between pre-specified and reported outcomes. Funnel plots were planned for outcomes with at least ten studies.

Data Synthesis and Statistical Analysis

Meta-analyses were performed using Review Manager (RevMan) version 5.1. For dichotomous outcomes, results were expressed as risk ratios (RRs) with 95% CIs. For continuous outcomes, MDs with 95% CIs were calculated. A random-effects model was used to account for expected clinical and methodological heterogeneity across studies.

Statistical heterogeneity was assessed using the Chi-square test and quantified using the I² statistic, with values below 50% considered low, 50-74% moderate, and ≥75% high heterogeneity. Subgroup and sensitivity analyses were conducted where appropriate to explore sources of heterogeneity.

Sensitivity Analysis

Sensitivity analyses were conducted to examine the robustness of pooled estimates. This included excluding studies with a high risk of bias and performing leave-one-out analyses, whereby each study was sequentially removed to assess its influence on the overall effect estimates.

Certainty of Evidence

The certainty of evidence for each outcome was evaluated using the Grading of Recommendations Assessment, Development and Evaluation (GRADE) approach [21]. Evidence was rated as high, moderate, low, or very low certainty based on risk of bias, inconsistency, indirectness, imprecision, and publication bias. A summary of findings table (Table 3) was generated using GRADEpro GDT software.

**Table 3:** GRADE table showing the impact of nutrition interventions during pregnancy on maternal and neonatal outcomes The risk in the intervention group (with its 95% CI) was calculated based on the assumed risk in the comparison group and the relative effect of the intervention (with its 95% CI). Patient or population: Pregnant women Setting: Community Intervention: Nutrition supplements Comparison: Placebo or standard care MD: mean difference; RR: risk ratio; RCT: randomized controlled trial: GRADE: Grading of Recommendations Assessment, Development and Evaluation. GRADE Working Group grades of evidence High certainty: We are very confident that the true effect lies close to the estimated effect. Moderate certainty: We are moderately confident in the effect estimate; the true effect is likely to be close to the estimated effect, but there is a possibility that it may be substantially different. Low certainty: Our confidence in the effect estimate is limited; the true effect may be substantially different from the estimated effect. Very low certainty: We have very little confidence in the effect estimate; the true effect is likely to be substantially different from the estimated effect. Explanations a. One trial was open label; b. extreme heterogeneity was observed in some pooled analyses; c. some trials were conducted using an open-label design; d. the 95% confidence interval was very wide for certain outcomes; e. methods of blinding were not clearly described in some studies; f. several outcomes had wide 95% confidence intervals; and g. heterogeneity was high across specific analyses.

Outcome (No. of participants (N), No. of studies)	Mean difference or relative risk (95% CI)	Anticipated absolute effects (95% CI)			
		Nutrition supplements	Placebo or standard care	Difference	Certainty of evidence
Maternal weight gain (g) (N: 1560; 2 RCTs)	MD 60.34 (59.97 to 60.71)	Mean pre-pregnancy weight: 4690	Mean pre-pregnancy weight: 4675	60.34 higher (59.97 higher to 60.71 higher)	⨁◯◯◯ Very low^a,b^
Birth weight (g) (N: 3236; 5 RCTs)	MD 48.54 (1.28 to 95.81)	Mean birth weight: 2752.32	Mean birth weight: 2749.66	48.54 higher (1.28 higher to 95.81 higher)	⨁◯◯◯ Very low^c,d^
Birth length (cm) (N: 3265; 4 RCTs)	MD 0.27 (0.12 to 0.42)	Mean birth length: 47.9	Mean birth length: 47.4	0.27 higher (0.12 higher to 0.42 higher)	⨁⨁⨁◯ Moderate^c^
Head circumference (cm) (N: 3224; 4 RCTs)	MD -0.52 (-0.63 to -0.42)	Mean head circumference: 33.76	Mean head circumference: 33.87	0.52 lower (0.63 lower to 0.42 lower)	⨁◯◯◯ Very low^b,c^
Maternal death (N: 1701; 2 RCTs)	RR 2.3 (0.34 to 15.45)	0.3% (0 to 1.8)	0.10%	0.2% more (0.1 fewer to 1.7 more)	⨁◯◯◯ Very low^a,d^
Stillbirth (N: 4096; 4 RCTs)	RR 0.95 (0.81 to 1.12)	11.3% (9.6 to 13.2)	11.70%	0.1% fewer (2.1 fewer to 1.5 more)	⨁⨁⨁◯ Moderate^c^
Preterm delivery (N: 1192; 2 RCTs)	RR 1.23 (0.38 to 3.94)	13.5% (4.2 to 43.2)	11.00%	2.5% more (6.8 fewer to 32.3 more)	⨁◯◯◯ Very low^a,b,d^
Neonatal death (N: 2169; 2 RCTs)	RR 0.63 (0.39 to 1.02)	2.5% (1.5 to 4)	3.90%	1.4% fewer (2.4 fewer to 0.1 more)	⨁⨁◯◯ Low^e,f^
Low birth weight (LBW) (N: 3239; 3 RCTs)	RR 0.75 (0.64 to 0.87)	26.5% (21.2 to 32.7)	31.20%	4.7% fewer (10 fewer to 1.6 more)	⨁⨁◯◯ Low^c,g^
Miscarriage (N: 1711; 3 RCTs)	RR 0.81 (0.48 to 1.37)	2.7% (1.6 to 4.6)	3.40%	0.6% fewer (1.8 fewer to 1.3 more)	⨁⨁◯◯ Low^c,f^

Results

Data and Analysis

A total of 14 maternal and neonatal outcomes were assessed from the included studies. For each outcome, effect estimates with 95% CIs are presented, along with the corresponding forest plots (Table 4).

**Table 4:** Protein-energy supplementation versus standard care. Note: The table is original and was prepared by the authors based on the included studies.

Outcome or subgroup	Studies	Participants	Statistical method	Effect estimate
1.1 Birth weight	5	3236	Mean Difference (IV, Fixed, 95% CI)	48.54 [1.28, 95.81]
1.2 Birth length	4	3265	Mean Difference (IV, Fixed, 95% CI)	0.27 [0.12, 0.42]
1.3 Head circumference	4	3224	Mean Difference (IV, Fixed, 95% CI)	-0.52 [-0.63, -0.42]
1.4 BMI of pregnant women	1	1640	Mean Difference (IV, Fixed, 95% CI)	0.08 [-0.04, 0.20]
1.5 MUAC	2	1535	Mean Difference (IV, Fixed, 95% CI)	0.09 [0.01, 0.17]
1.6 Small for gestational age (SGA)	1	976	Risk Ratio (M-H, Fixed, 95% CI)	0.87 [0.74, 1.02]
1.7 Stillbirth	4	4096	Risk Ratio (M-H, Fixed, 95% CI)	0.95 [0.81, 1.12]
1.8 Proportion of low birth weight (LBW) babies	3	3239	Risk Ratio (M-H, Fixed, 95% CI)	0.75 [0.64, 0.87]
1.9 Preterm birth	2	1192	Risk Ratio (M-H, Random, 95% CI)	1.23 [0.38, 3.94]
1.10 Miscarriage	3	1711	Risk Ratio (M-H, Fixed, 95% CI)	0.81 [0.48, 1.37]
1.11 Neonatal death	2	2169	Risk Ratio (M-H, Fixed, 95% CI)	0.63 [0.39, 1.02]
1.12 Gestational age	2	296	Mean Difference (IV, Fixed, 95% CI)	-0.50 [-1.01, -0.00]
1.13 Gestational weight gain	2	1560	Mean Difference (IV, Fixed, 95% CI)	60.34 [59.97, 60.71]
1.14 Maternal death	2	1701	Risk Ratio (M-H, Fixed, 95% CI)	2.30 [0.34, 15.45]

Primary Outcomes

A total of 14 maternal and neonatal outcomes were assessed. Protein-energy supplementation significantly increased mean birth weight (five studies; n = 3,236; MD 48.54 g, 95% CI 1.28 to 95.81) [16-20] and birth length (four studies; n = 3,265; MD 0.27 cm, 95% CI 0.12 to 0.42) [16-20]. Head circumference was slightly lower in the intervention group (four studies; n = 3,224; MD -0.52 cm, 95% CI -0.63 to -0.42) [16-20]. Gestational weight gain was significantly higher among supplemented women (two studies; n = 1,560; MD 60.34 g, 95% CI 59.97 to 60.71) [19,20], whereas BMI did not differ significantly between groups (one study; n = 1,640; MD 0.08 kg/m², 95% CI -0.04 to 0.20) [17].

**Figure 3:**
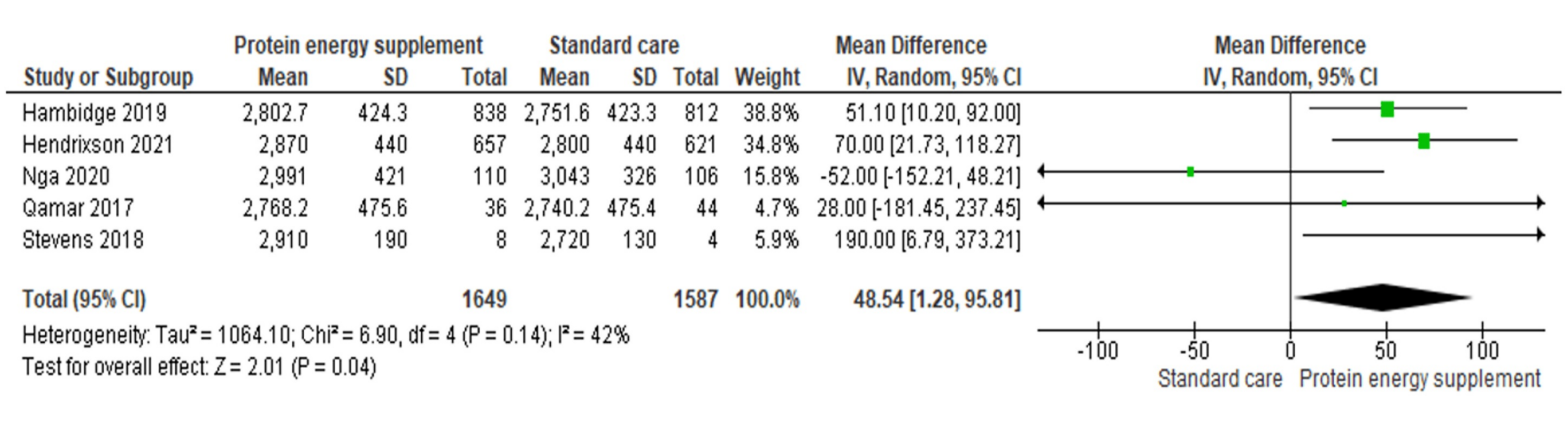
Forest plot of comparison 1: protein-energy supplementation versus standard care, outcome 1.1: birth weight. Source: Hambidge KM, et al. (2019) [17]; Hendrixson DT, et al. (2020) [20]; Nga HT, et al. (2020) [19]; Qamar FN, et al. (2017) [16]; and Stevens B, et al. (2018) [18]. This figure was prepared by the authors for the present systematic review and meta-analysis.

Birth weight: Five RCTs involving 3,236 participants demonstrated that protein-energy supplementation significantly increased mean birth weight compared with standard care (MD 48.54 g, 95% CI 1.28 to 95.81) (Figure 3) [16-20].

**Figure 4:**
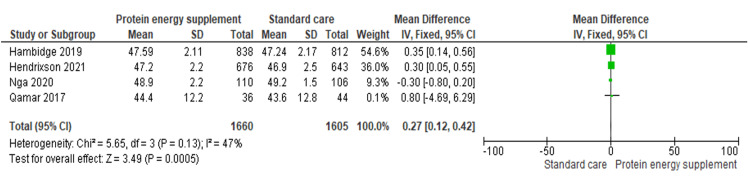
Forest plot of comparison 1: protein-energy supplementation vs standard care, outcome 1.2: birth length. Source: Hambidge KM, et al. (2019) [17]; Hendrixson DT, et al. (2020) [20]; Nga HT, et al. (2020) [19]; and Qamar FN, et al. (2017) [16]. All figures included in this systematic review and meta-analysis were prepared by the authors and are original.

Birth length: Four trials including 3,265 participants showed a significant improvement in birth length among women receiving protein-energy supplementation (MD 0.27 cm, 95% CI 0.12 to 0.42) (Figure 4) [16,17,19,20].

**Figure 5:**
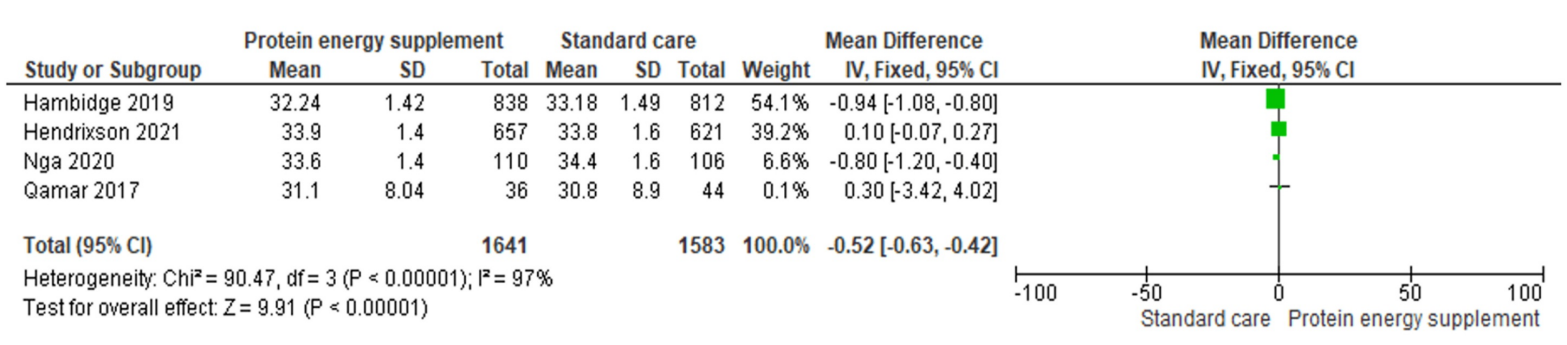
Forest plot of comparison 1: protein-energy supplementation vs standard care, outcome 1.3: head circumference. Source: Hambidge KM, et al. (2019) [17]; Hendrixson DT, et al. (2020) [20]; Nga HT, et al. (2020) [19]; and Qamar FN, et al. (2017) [16]. All figures included in this systematic review and meta-analysis were prepared by the authors and are original.

Head circumference: Four studies (n = 3,224) reported a statistically significant reduction in mean head circumference with protein-energy supplementation (MD = -0.52 cm, 95% CI: -0.63 to -0.42). Unexpectedly, protein-energy supplementation was associated with a slight decrease in head circumference. The reduction is small, and its clinical relevance is uncertain. It may reflect variation in fetal growth patterns or measurement error rather than a true adverse effect (Figure 5) [16,17,19-20].

**Figure 6:**
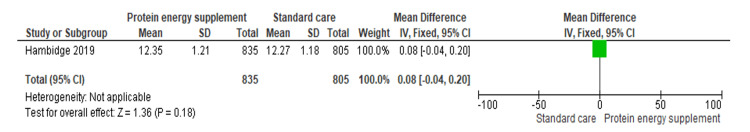
Forest plot of comparison 1: protein-energy supplementation vs standard care, outcome 1.4: BMI. Source: Hambidge KM, et al. (2019) [17]. All figures included in this systematic review and meta-analysis were prepared by the authors and are original.

BMI: BMI during pregnancy, assessed in one large trial (n = 1,640), showed no significant difference between the intervention and control groups (MD 0.08 kg/m², 95% CI -0.04 to 0.20) (Figure 6) [17].

**Figure 7:**
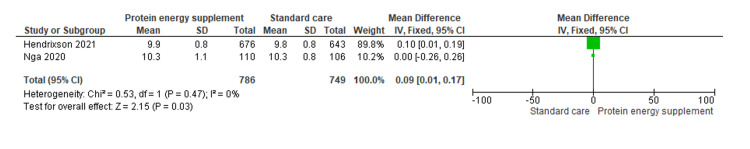
Forest plot of comparison 1: protein-energy supplementation vs standard care, outcome 1.5: MUAC Source: Hendrixson DT, et al. (2020) [20] and Nga HT, et al. (2020) [19]. All figures included in this systematic review and meta-analysis were prepared by the authors and are original.

Mid-upper arm circumference (MUAC): Two studies (n = 1,535) indicated a significant improvement in MUAC with protein-energy supplementation (MD 0.09 cm, 95% CI: 0.01-0.17). Protein-energy supplementation modestly increased MUAC [19-20]. MUAC is a key marker of nutritional status; even small gains can have implications for infant survival in resource-limited settings (Figure 7).

**Figure 8:**
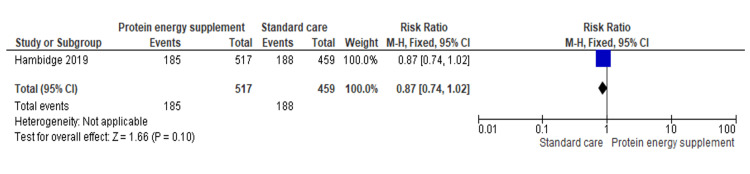
Forest plot of comparison 1: protein-energy supplementation vs standard care, outcome 1.6: small for gestational age (SGA). Source: Hambidge KM, et al. (2019) [17]. All figures included in this systematic review and meta-analysis were prepared by the authors and are original.

Small for gestational age (SGA): One study (n = 976) found no significant reduction in SGA risk (RR = 0.87, 95% CI: 0.74-1.02). Protein-energy supplementation showed a non-significant trend towards reducing SGA births. Although not statistically significant, the direction of effect suggests potential benefit that may be more evident in larger samples (Figure 8) [17].

**Figure 9:**
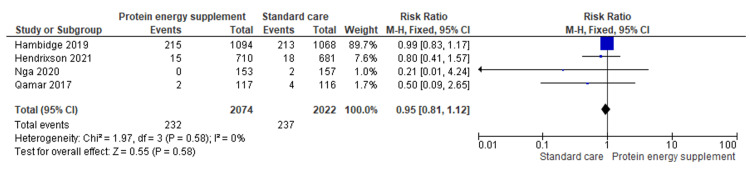
Forest plot of comparison 1: protein-energy supplementation vs standard care, outcome 1.7: stillbirth. Source: Hambidge KM, et al. (2019) [17]; Hendrixson DT, et al. (2020) [20]; Nga HT, et al. (2020) [19]; and Qamar FN, et al. (2017) [16]. All figures included in this systematic review and meta-analysis were prepared by the authors and are original.

Stillbirth: The pooled analysis of four studies revealed no significant difference between the intervention and control groups (RR = 0.95; 95% CI: 0.81-1.12; Z = 0.55; p = 0.58), indicating that protein-energy supplementation had no measurable effect on the risk of the outcome compared with standard care (Figure 9) [16,17,19,20].

**Figure 10:**
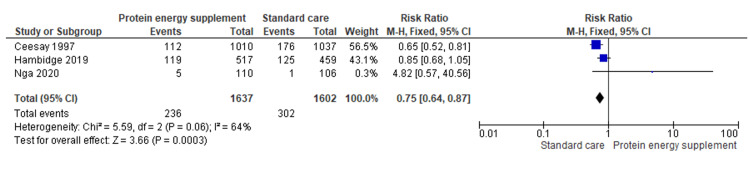
Forest plot of comparison 1: protein-energy supplementation vs standard care, outcome 1.8: proportion of LBW babies. LBW: Low birth weight. Source: Ceesay SM, et al. (1997) [15]; Hambidge KM, et al. (2019) [17]; and Nga HT, et al. (2020) [19]. All figures included in this systematic review and meta-analysis were prepared by the authors and are original.

Low birth weight (LBW): The pooled analysis showed a statistically significant reduction in low birth weight among participants receiving the intervention compared with usual care (RR = 0.75; 95% CI: 0.64-0.87; Z = 3.66; p = 0.0003), corresponding to an approximately 25% lower risk of low birth weight in the intervention group (Figure 10) [15,17,19].

**Figure 11:**
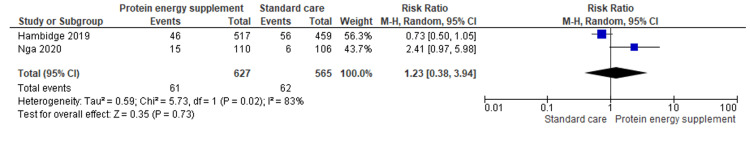
Forest plot of comparison 1: protein-energy supplementation vs standard care, outcome 1.9: preterm birth. Source: Hambidge KM, et al. (2019) [17] and Nga HT, et al. (2020) [19]. All figures included in this systematic review and meta-analysis were prepared by the authors and are original.

Preterm birth: Across two studies (n = 1,192), no significant difference in preterm birth risk was observed (RR = 1.23, 95% CI: 0.38-3.94). No evidence suggests that the intervention affects the risk of preterm delivery. Nutritional support may need to be combined with other interventions to address preterm birth (Figure 11) [17,19].

**Figure 12:**
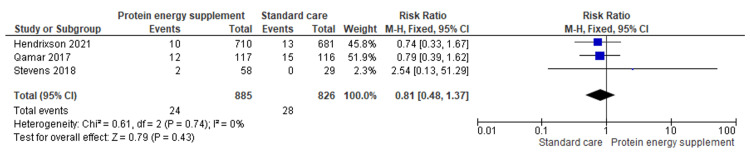
Forest plot of comparison 1: protein-energy supplementation vs standard care, outcome 1.10: miscarriage. Source: Hambidge KM, et al. (2019) [17]; Qamar FN, et al. (2017) [16]; and Stevens B, et al. (2018) [18]. All figures included in this systematic review and meta-analysis were prepared by the authors and are original.

Miscarriage: Analysis of three studies (n = 1,711) showed that the intervention was not significantly associated with miscarriage risk (RR = 0.81; 95% CI: 0.48-1.37). No measurable effect on miscarriage rates was observed, likely reflecting the influence of multiple non-nutritional factors that limit the impact of nutrition alone on this outcome (Figure 12) [16-18].

**Figure 13:**
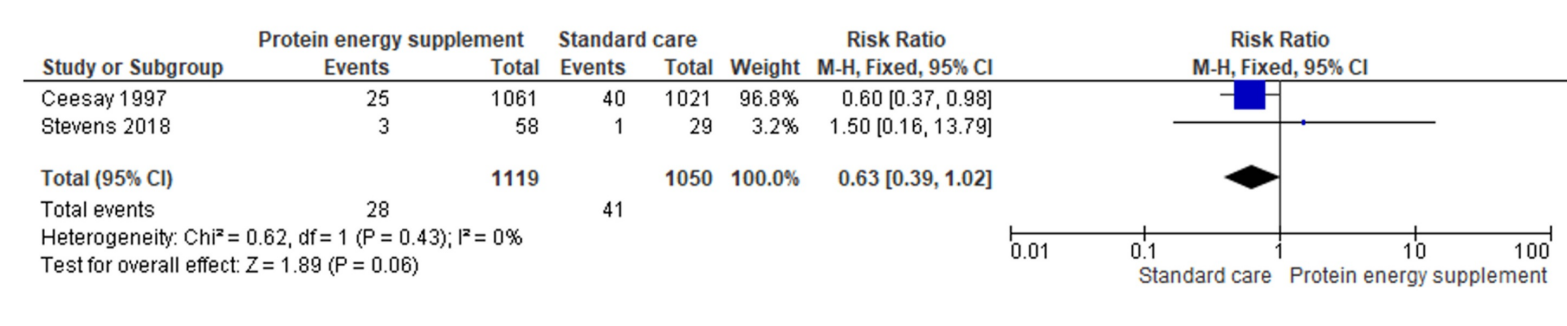
Forest plot of comparison 1: protein-energy supplementation vs standard care, outcome 1.11: neonatal death. Source: Ceesay SM, et al. (1997) [15] and Stevens B, et al. (2018) [18]. All figures included in this systematic review and meta-analysis were prepared by the authors and are original.

Neonatal death: Analysis of two studies (n = 2,169) indicated a trend toward lower neonatal mortality with the intervention (RR = 0.63, 95% CI: 0.39-1.02), although the difference was not statistically significant, it was clinically relevant. Even modest reductions in neonatal mortality are important in public health terms, warranting further research (Figure 13) [15,18].

**Figure 14:**
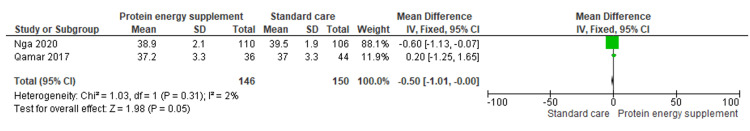
Forest plot of comparison 1: protein-energy supplementation vs standard care, outcome 1.12: gestational age. Source: Nga HT, et al. (2020) [19] and Qamar FN, et al. (2017) [16]. All figures included in this systematic review and meta-analysis were prepared by the authors and are original.

Gestational age: Two studies (n = 296) reported a small but statistically significant reduction in gestational age in the intervention group (MD = -0.50 weeks, 95% CI: -1.01 to -0.00). The intervention was associated with slightly shorter gestation. The difference is minimal and unlikely to be clinically meaningful, but warrants monitoring in future studies (Figure 14) [16,19].

**Figure 15:**
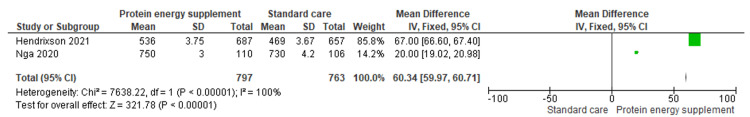
Forest plot of comparison 1: protein-energy supplementation vs standard care, outcome 1.13: gestational weight gain. Source: Hendrixson DT, et al. (2020) [20] and Nga HT, et al. (2020) [19]. All figures included in this systematic review and meta-analysis were prepared by the authors and are original.

Gestational weight gain: Two studies (n = 1,560) reported that protein-energy supplementation significantly increased gestational weight gain compared with standard care (MD 60.34 g, 95% CI 59.97 to 60.71) (Figure 15) [19,20].

**Figure 16:**
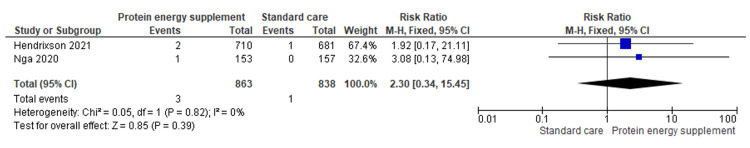
Forest plot of comparison 1: protein-energy supplementation vs standard care, outcome 1.14: maternal death. Source: Hendrixson DT, et al. (2020) [20] and Nga HT, et al. (2020) [19]. All figures included in this systematic review and meta-analysis were prepared by the authors and are original.

Maternal death: Analysis of two studies (n = 1,701) indicated no statistically significant difference in the risk of maternal mortality (RR = 2.30, 95% CI: 0.34-15.45). No effect on maternal death was detected, with wide CIs indicating uncertainty. Protein-energy supplementation alone is unlikely to influence maternal mortality without broader maternal health strategies (Figure 16) [19-20].

Discussion

This systematic review analyzed data from RCTs conducted primarily in low- and middle-income countries, including Gambia, Pakistan, Bangladesh, Vietnam, Sierra Leone, and the multi-country Women First Trial implemented in India, the Democratic Republic of the Congo, Guatemala, and Pakistan. Across diverse geographic and nutritional contexts, balanced protein-energy supplementation during pregnancy demonstrated modest but directionally consistent improvements in maternal gestational weight gain and neonatal anthropometric outcomes, particularly birth weight and birth length. In contrast, effects on perinatal mortality and major obstetric complications were generally small and statistically non-significant.

When the findings are considered collectively, a coherent pattern emerges. Improvements in maternal energy intake were consistently associated with measurable gains in fetal growth parameters [15]. Trials providing higher caloric supplementation during nutritionally vulnerable periods reported improvements in birth outcomes among chronically undernourished women, while studies initiating supplementation earlier, including during preconception phases, suggested potential benefits for fetal linear growth [17]. Together, these findings indicate that both the dosage and timing of supplementation influence effectiveness. Acute energy deficits may be mitigated by higher caloric intake during pregnancy, whereas earlier nutritional optimization may affect placental development and long-term fetal growth trajectories.

The majority of included trials enrolled nutritionally vulnerable women characterized by low BMI or reduced mid-upper arm circumference. Greater benefits were generally observed in these populations, suggesting that baseline maternal undernutrition modifies intervention effectiveness. This supports the biological plausibility that supplementation yields the greatest benefit when correcting pre-existing nutritional deficits [16].

Although neonatal anthropometric gains were modest, they translated into a meaningful reduction in low birth weight. Even small increases in mean birth weight at the population level may substantially reduce the proportion of infants falling below clinically significant thresholds [18]. However, supplementation did not consistently reduce stillbirth, miscarriage, preterm birth, or maternal mortality [19]. These outcomes are influenced by multifactorial determinants, including infection, hypertensive disorders, obstetric complications, and access to quality health services. The consistency of null findings across heterogeneous settings suggests that balanced protein-energy supplementation primarily influences growth-mediated outcomes rather than acute mortality-related pathways.

The observed small reduction in head circumference requires cautious interpretation. The absolute difference was minimal and occurred alongside improvements in birth weight and length, making a biologically adverse effect unlikely. Measurement variability and methodological differences across studies may partially explain this finding.

Heterogeneity among trials likely reflects variation in caloric content, protein composition, timing of initiation, supplement formulation, the presence of co-interventions, and baseline nutritional status. Despite these differences, the direction of effect on birth weight and length remained largely consistent, strengthening confidence in a causal relationship between maternal nutritional support and fetal growth [21-23].

From a public health perspective, balanced protein-energy supplementation represents a pragmatic strategy within antenatal care programs in undernourished populations. However, supplementation should not be viewed as a standalone solution. Integration with micronutrient supplementation, infection prevention strategies, and high-quality antenatal care is essential to achieve broader reductions in maternal and neonatal morbidity and mortality. Addressing structural determinants of food insecurity and poverty remains fundamental for sustained impact.

The strengths of this review include the exclusive inclusion of RCTs, representation of community-based implementation settings, and geographic diversity across Africa and Asia. Limitations include heterogeneity in intervention composition, limited blinding in open-label designs, attrition in some studies, and insufficient statistical power for rare outcomes such as maternal mortality. Additionally, the predominance of studies conducted in undernourished populations may limit generalizability to well-nourished settings.

Future research should prioritize adequately powered trials with standardized supplementation protocols, clear reporting of caloric and protein composition, and evaluation of the timing of initiation. Comparative assessment of preconception versus antenatal supplementation and long-term follow-up examining child growth and developmental outcomes would strengthen the evidence base. Implementation research addressing adherence, cost-effectiveness, and integration into routine health systems is also warranted.

## Conclusions

Balanced protein energy supplementation during pregnancy provides modest but meaningful benefits for maternal gestational weight gain and neonatal growth outcomes, particularly birth weight and birth length, among populations affected by undernutrition. However, the evidence does not demonstrate consistent effects on perinatal morbidity or mortality outcomes, highlighting the multifactorial nature of adverse pregnancy events.

These findings support the inclusion of nutrition supplementation as a complementary component of antenatal care in resource-limited settings, especially where maternal undernutrition and low birth weight are prevalent. To achieve broader improvements in maternal and neonatal health, supplementation strategies should be implemented alongside comprehensive maternal health interventions. Further high-quality, context-specific trials are needed to refine intervention design, strengthen the certainty of evidence, and guide effective scale-up.
